# Effect on Rotation Speed on Thermal Dehydration Characteristics of Waste Gypsum Particles in a Constant Volume Rotary Vessel by Heating

**DOI:** 10.3390/ma17061276

**Published:** 2024-03-10

**Authors:** Koichiro Ogata, Kotetsu Arimura, Hayate Gotoh, Kai Satoh, Kazuki Tokumaru, Hideo Kawahara, Hiroaki Sano

**Affiliations:** 1 Department of Mechanical Engineering, National Institute of Technology, Oita College, 1666 Maki, Oita 870-0152, Japank-tokumaru@oita-ct.ac.jp (K.T.); 2 Department of Mechanical Systems Engineering, National Defense Academy, 1-10-20 Hashirimizu, Yokosuka 239-8686, Japan; kawahara@nda.ac.jp; 3 Department of Civil and Environmental Engineering, National Defense Academy, 1-10-20 Hashirimizu, Yokosuka 239-8686, Japan; sano@nda.ac.jp

**Keywords:** waste gypsum, constant-volume rotary vessel, heating, rotation speed, thermal dehydration

## Abstract

This study examined the thermal dehydration characteristics of CaSO_4_∙2H_2_O in a constant-volume rotary vessel. The experiment used CaSO_4_∙2H_2_O particles obtained from the crushed waste gypsum board. The particle size ranged from 850 to 2000 μm, and the experiment was carried out at varying rotation speeds of 1, 10, and 35 rpm, with the vessel temperature heated to 180 °C. Temperature and pressure inside the vessel were measured simultaneously using the thermocouple and the pressure sensor. The XRPD measurement analyzed the transition of CaSO_4_∙2H_2_O after the heating of particles. The result showed that the temperature growth rate was similar for high rotation speeds of 10 and 35 rpm, while periodic temperature changes occurred at the low rotation speed of 1 rpm. A distinguishing flow pattern was observed at the low rotation speed, and the particles inside the vessel collapsed periodically downward. This particle behavior was related to the temperature distribution of the rotation speed of 1 rpm. Additionally, the pressure in the vessel increased rapidly at higher rotation speeds. This trend indicates the desorption of the crystal water of CaSO_4_∙2H_2_O due to the increasing temperature in the case of high rotation speed. Also, the XRPD measurement results showed the appearance of CaSO_4_∙0.5H_2_O under the higher rotation speed conditions, and the mass fraction of CaSO_4_∙0.5H_2_O increased with the rotation speed. Overall, the present study suggests that rotation speed plays a crucial role in determining the heat conduction and heat transfer of particles in a constant-volume rotary vessel.

## 1. Introduction

The gypsum board is made of a gypsum core material and is covered on both sides with base paper to create a flat panel. This type of board is known for its excellent fire resistance, sound insulation, heat insulation, and workability, making it an ideal building material. Additionally, it is great to see that flue gas desulfurization gypsum and wastepaper are also used as raw materials for gypsum boards, making them excellent recycled materials. Due to these advantages, the gypsum board is extremely popular as an interior base material for building walls, floors, and ceilings, and its production volume is increasing in Japan.

On the other hand, the quantity of waste gypsum boards generated from ageing buildings is increasing significantly. Unfortunately, most of these waste gypsum boards are disposed of in landfills. As a result, it is now considered controlled industrial waste due to instances where hydrogen sulfide has been produced in final disposal sites that handle waste gypsum boards in various locations. Consequently, processing waste gypsum boards has become more expensive [1]. Under these circumstances, ongoing efforts are being made to convert CaSO_4_∙2H_2_O produced from waste gypsum boards into CaSO_4_∙0.5H_2_O, which solidifies after being mixed with water and is used as a ground improvement material [2,3,4,5,6,7,8,9].

Generally, gypsum is transferred from CaSO_4_∙2H_2_O to CaSO_4_∙0.5H_2_O or CaSO_4_ [10]. Research is also underway on the conversion of gypsum on Mars [11]. It is well known that a heating device converts CaSO_4_∙2H_2_O to CaSO_4_∙0.5H_2_O or CaSO_4_. Different heating devices such as rotary kilns [2] and electric furnaces [3,4] are commonly used, while far-infrared-type [5] devices have also been developed. For example, the gypsum is rotated and dispersed inside the kiln and dried using radiant heat from the burner flame and hot air in the case of a rotary kiln [2]. In addition, CaSO_4_∙2H_2_O, discharged by separating and crushing waste gypsum boards into paper and gypsum, has a broad particle size distribution ranging from 2 mm or less to several tens of micrometers. Therefore, it is crucial to treat gypsum boards via temperature management and heating control during the manufacturing process of CaSO_4_∙0.5H_2_O [4].

Since CaSO_4_∙0.5H_2_O solidifies with water, it is ideal to convert all CaSO_4_∙2H_2_O to CaSO_4_∙0.5H_2_O from the perspective of reusing gypsum. However, it has been pointed out that non-uniform heating of CaSO_4_∙2H_2_O in the heating device occurs due to a large amount of CaSO_4_∙2H_2_O being heated simultaneously [5,7]. For this purpose, equipment using rotary kiln heating and a reacting tank have been developed [2]. Although this method has improved the production rate of CaSO_4_∙0.5H_2_O, some CaSO_4_∙2H_2_O remains after heat treatment [2]. This is because CaSO_4_∙2H_2_O that is handled has a broad particle size distribution. Additionally, the drying characteristics of gypsum due to the heating applied to CaSO_4_∙2H_2_O are unclear. Furthermore, the conversion characteristics of CaSO_4_∙2H_2_O to CaSO_4_∙0.5H_2_O have yet to be elucidated when temperature and pressure change.

This study evaluated the conversion characteristics of CaSO_4_∙2H_2_O produced from waste gypsum boards to CaSO_4_∙0.5H_2_O. The test equipment used was a closed constant-volume rotary heating device to vary the temperature, pressure, and rotational speed. This paper discusses the results of investigating the thermal dehydration properties of CaSO_4_∙2H_2_O when the particle size and initial filling mass of gypsum are kept constant, and the heating temperature, pressure, and rotational speed inside the vessel are varied.

## 2. Experiment

### 2.1. Experimental Equipment

**Figure 1:**
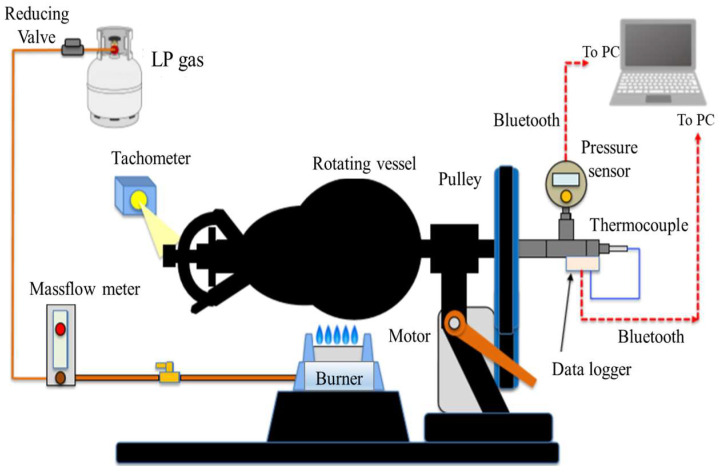
Material heating equipment using a constant-volume rotary vessel.

Figure 1 shows this study’s schematic diagram of constant-volume rotary heating equipment. The rotating vessel in the figure has an elliptical shape, and the sealed state is achieved by closing the pressure valve and sample insertion port. In the experiment, CaSO_4_∙2H_2_O was naturally filled into a vessel, which was sealed and heated while rotating. LP gas was used as the heating source. The rotational motion of the rotating vessel was provided using a motor, pulley, and belt. A thermocouple (Hakko Denki, Nagano, Japan) and a pressure sensor (Krone, KDM30, Tokyo, Japan) were used to measure the temperature and pressure inside the rotating vessel. The temperature and pressure data sampling frequency are 1 s and 1.98 s, respectively. The sampling frequency difference depends on the different types of data loggers used to measure the temperature and the pressure. In this measurement, the beginning of the recording time for these sensors was the same. Then, we could obtain the time history data of the temperature and the pressure inside the rotating vessel. In addition, qualitative and quantitative analyses were performed using an X-ray powder diffraction device (Rigaku, MiniFlex 600, Tokyo, Japan) to investigate the conversion state of gypsum after heating.

### 2.2. Particles Used and Experimental Conditions

The particles used in this study were CaSO_4_∙2H_2_O derived from a waste gypsum board, with a particle size of less than 2000 μm, collected from industrial waste treatment facilities in Okinawa Prefecture. In this study, the raw powder of CaSO_4_∙2H_2_O was sieved to adjust the particle size to a range from 850 to 2000 μm. The material density was 2376 kg/m^3^.

The volume of the elliptical rotating container shown in Figure 1 was 1450 cm^3^. In this study, the initial filling mass of powder was 100 g. The fuel flow rate was set to 1.2 L/min. Heating experiments were conducted with the rotation speed set at 1, 10, and 35 rpm.

## 3. Results

### 3.1. Conversion Characteristics of Gypsum

**Figure 2:**
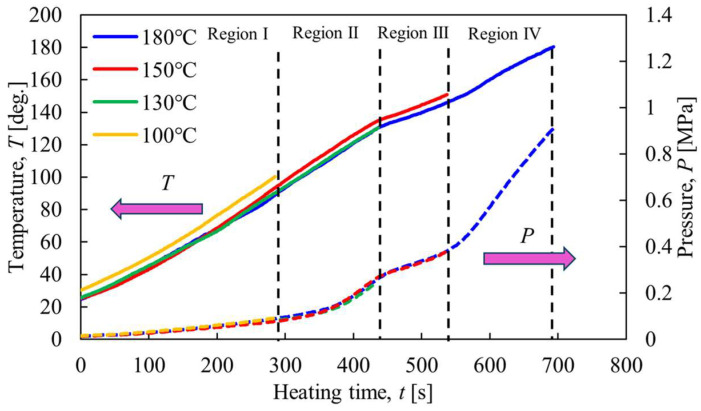
Time histories of temperature and pressure inside the rotary vessel.

Figure 2 shows an example of the results of a heating experiment using constant-volume rotary heating equipment. The first vertical axis is the temperature inside the vessel, the second is the gauge pressure, and the horizontal axis is the heating time. The initial filling mass of particles was 100 g, the rotation speed was 35 rpm, and the heating end temperature was set to 100, 130, 150, and 180 °C. The figure shows that the slope of temperature and pressure against heating time is almost constant until around 300 s in region I. It is thought that an increase in internal energy due to heating occurred in this region. Next, in region II in the figure, the temperature gradient is almost constant as in region I, but there is a tendency for the pressure to increase from around 350 s. The temperature at this time was around 110 °C, and the pressure was increased. It is inferred that steam was generated in the vessel due to the desorption of crystallized water from CaSO_4_∙2H_2_O [12]. After that, in region III, from 450 s to around 530 s, the slopes of temperature and pressure concerning time are smaller than in region II. Since the internal temperature of the vessel in this region is between 130 °C and 150 °C, it is assumed that evaporation of the crystallized water of CaSO_4_∙2H_2_O was active, and the state changed to CaSO_4_∙0.5H_2_O. When further heating continued, the temperature and pressure inside the vessel rose rapidly, and it appeared that the vessel transitioned to a superheated steam state.

**Figure 3:**
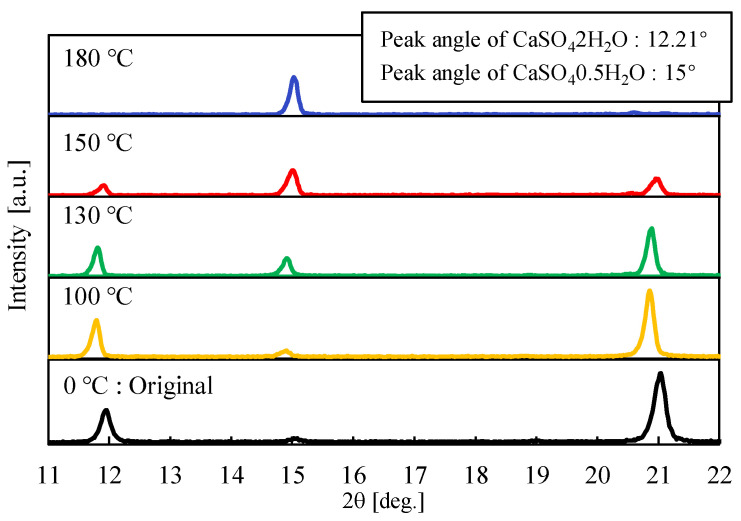
XRPD measurement results.

Figure 3 shows the results of the XRPD measurements. The peak intensities at 12° and 21° in the figure represent CaSO_4_∙2H_2_O, and the 15° peak represents CaSO_4_∙0.5H_2_O. Here, a temperature of 100 °C, 130 °C, 150 °C, and 180 °C corresponds to the results of regions I, II, III, and IV. The measurement result before heating indicates the original particles. As shown in the figure, the result of region I at a temperature of 100 °C maintains the state of CaSO_4_∙2H_2_O, similar to the original particles. When the temperature reaches 130 °C of region II, the peak intensity of CaSO_4_∙0.5H_2_O appears. When the temperature comes to region III of 150 °C, the peak intensity of CaSO_4_∙0.5H_2_O exceeds the intensity of CaSO_4_∙2H_2_O. Furthermore, it can be confirmed that only the peak of CaSO_4_∙0.5H_2_O appears when the heating temperature is up to 180 °C. As described above, using closed rotary heating equipment, this experiment could capture the conversion of CaSO_4_∙2H_2_O to CaSO_4_∙0.5H_2_O.

### 3.2. Effect of Rotation Speed on Conversion Characteristics of Gypsum

**Figure 4:**
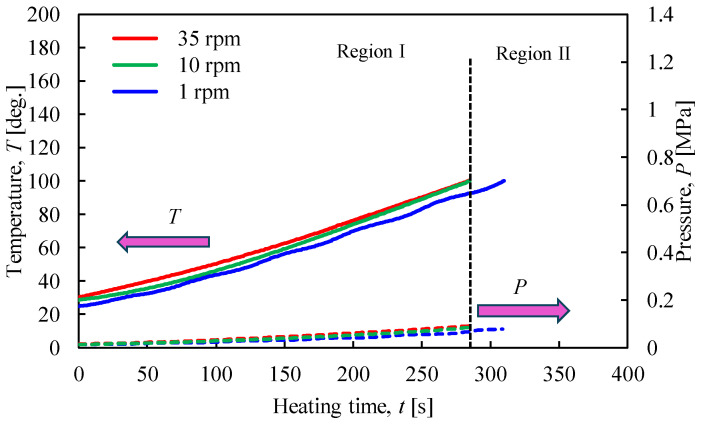
Time histories of temperature and pressure inside the rotary vessel at a heating end temperature of 100 °C when the rotation speed was changed.

**Figure 5:**
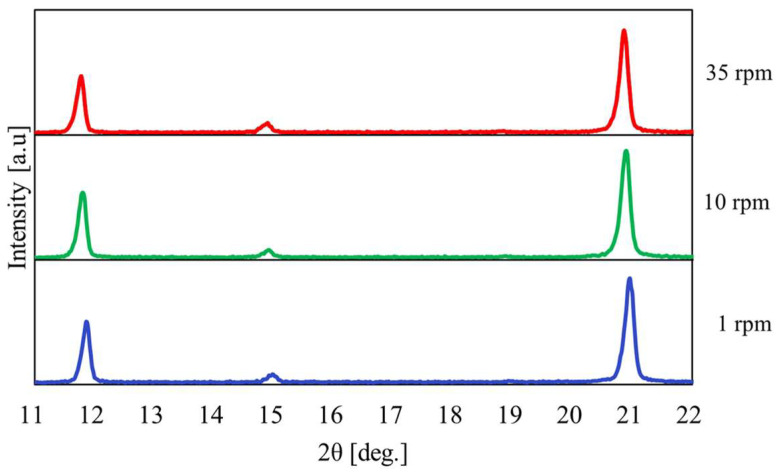
XRPD measurement results at a heating end temperature of 100 °C when the rotation speed was changed.

Figure 4 shows the relationship between the temperature and pressure inside the vessel against the heating time when the heating end temperature was 100 °C. In addition, Figure 5 indicates the XRPD measurement results where the rotation speed of the vessel was varied to 1, 10, and 35 rpm. Figure 4 shows that the temperature and pressure increased with a constant slope when the rotational speed was 10 and 35 rpm. On the other hand, it was confirmed that the temperature changed periodically under a rotation speed of 1 rpm. From the results in Figure 5, only the peak intensity of CaSO_4_∙2H_2_O appeared when the heating temperature was 100 °C, and no effect of change in rotation speed was observed.

**Figure 6:**
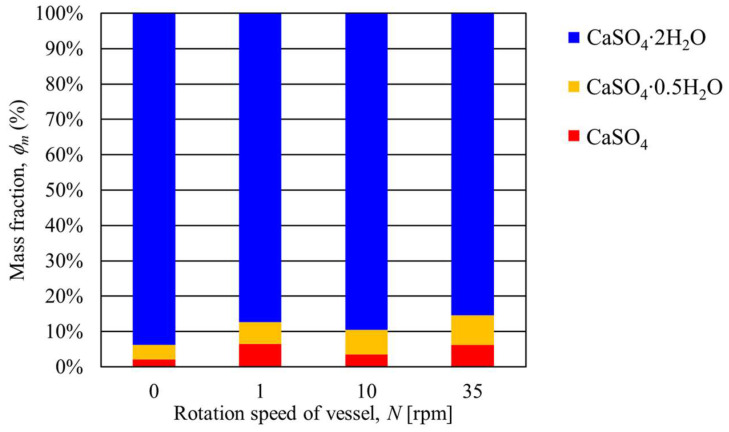
Relationship between the transferring mass fraction of gypsum and the rotation speed of the rotary vessel at a heating end temperature of 100 °C.

Table 1 shows the mass fraction of CaSO_4_∙2H_2_O, CaSO_4_∙0.5H_2_O, and CaSO_4_ at a heating end temperature of 100 °C and a rotation speed of 1, 10, and 35 rpm. Here, the mass fraction of each kind of gypsum was determined by Rietveld analysis. The conditions where the rotation speed and temperature are zero indicate the measurement data of the gypsum before heating. In addition, Figure 6 shows a graph comparing measured mass fractions organized by each rotation speed. As shown in the figure, when the heating end temperature was 100 °C, there was no significant difference in the mass fraction of gypsum even if the rotation speed changed. On the other hand, CaSO_4_∙2H_2_O before heating contained CaSO_4_∙0.5H_2_O and CaSO_4_. This is because the gypsum used in this study was a waste gypsum board, and it seems that these gypsums were generated during waste disposal.

**Figure 7:**
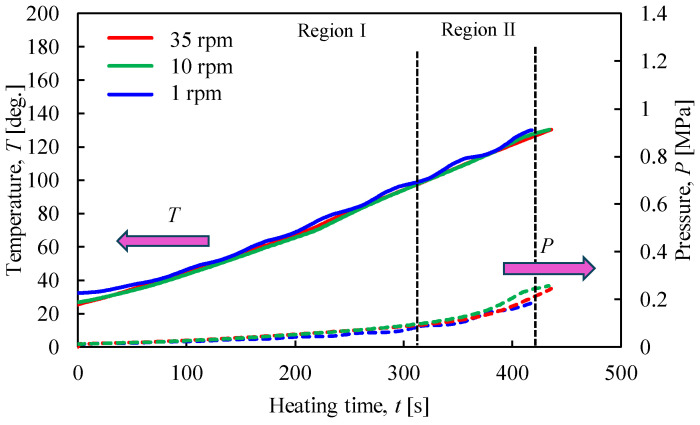
Time histories of temperature and pressure inside the rotary vessel at a heating end temperature of 130 °C when the rotation speed was changed.

**Figure 8:**
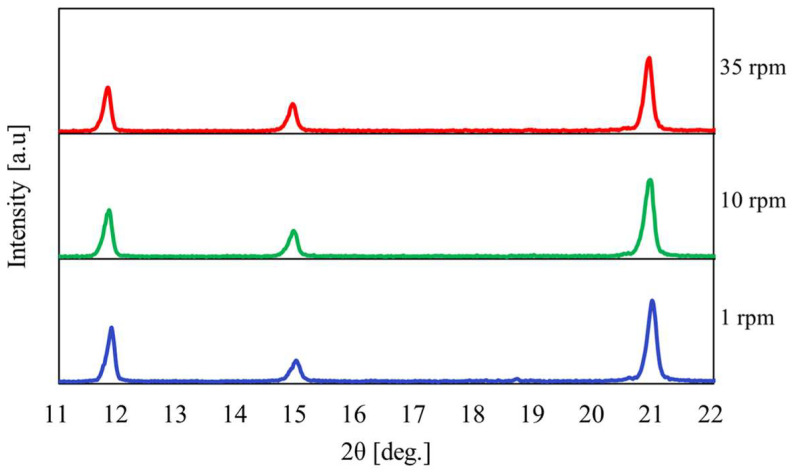
XRPD measurement results at a heating end temperature of 130 °C when the rotation speed was changed.

Figure 7 shows the relationship between the temperature and pressure inside the vessel when the heating end temperature was 130 °C. Figure 8 shows the results of the XRPD measurement. The rotation speed of the vessel was 1, 10, and 35 rpm. In Figure 7, the temperature gradient in region I appears almost constant when the rotation speed is 10 and 35 rpm. On the other hand, periodic changes in the temperature curve were confirmed under a rotation speed of 1 rpm. In region II, there was a tendency for the pressure to increase at all rotational speeds. From the XRPD measurement results in Figure 8, CaSO_4_∙0.5H_2_O can be confirmed at all rotation speeds. This result indicates that the desorption of crystallized water from the CaSO_4_∙2H_2_O has begun.

**Figure 9:**
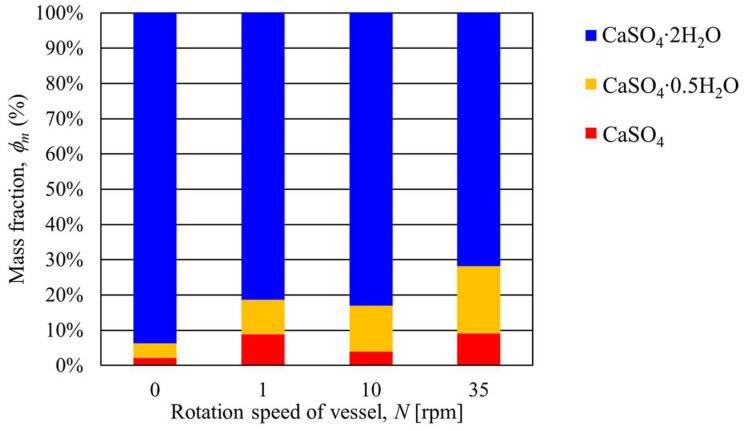
Relationship between the transferring mass fraction of gypsum and the rotation speed of the rotary vessel at a heating end temperature of 130 °C.

Table 2 shows the mass fractions of CaSO_4_∙2H_2_O, CaSO_4_∙0.5H_2_O, and CaSO_4_ under the conditions that the heating end temperature was 130 °C and the rotation speed was 1, 10, and 35 rpm. In addition, Figure 9 shows the mass fraction of gypsum organized at each rotation speed. The figure shows that the mass fraction of CaSO_4_∙2H_2_O at a rotation speed of 1 rpm decreased by 12.33% compared to without rotation. Additionally, the mass fraction of CaSO_4_∙0.5H_2_O increased by 5.63% under the same condition. Next, no significant change in the mass fractions of CaSO_4_∙2H_2_O can be confirmed between 1 rpm and 10 rpm rotation speeds. When the results of the rotation speeds of 1 rpm and 35 rpm were compared, the mass fraction of CaSO_4_∙2H_2_O at a rotation speed of 35 rpm decreased by 9.52% compared to the result of the rotation speed of 1 rpm. Furthermore, the mass fraction of CaSO_4_∙0.5H_2_O with a rotation speed of 35 rpm was 9.27% higher. These results show that although the amount is small, conversion from CaSO_4_∙2H_2_O to CaSO_4_∙0.5H_2_O begins when the heating end temperature is 130 °C.

**Figure 10:**
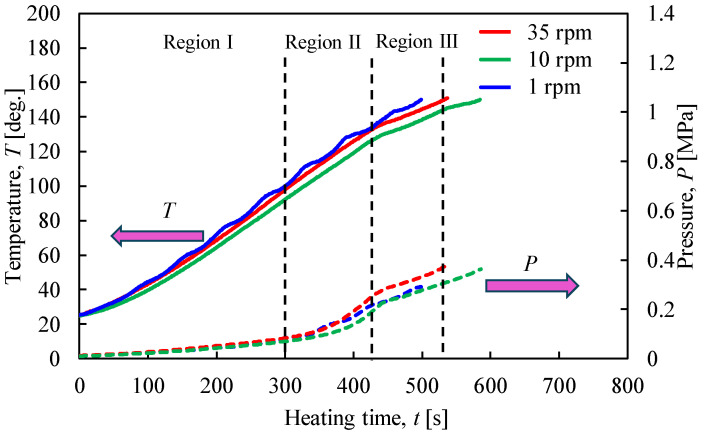
Time histories of temperature and pressure inside the rotary vessel at a heating end temperature of 150 °C when the rotation speed was changed.

**Figure 11:**
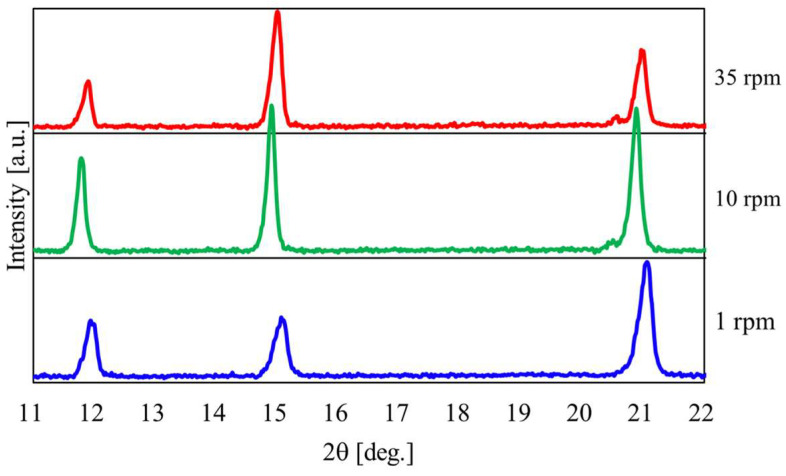
XRPD measurement results at a heating end temperature of 150 °C when the rotation speed was changed.

Figure 10 shows the relationship between the temperature and pressure inside the rotary vessel and the heating time when the heating end temperature was 150 °C. Figure 11 shows the XRPD measurement results. The rotation speed of the vessel was varied between 1, 10, and 35 rpm. From Figure 10, periodic temperature changes were confirmed when the rotation speed was 1 rpm, similar to the heating end temperatures of 100 °C and 130 °C. The slope of temperature and pressure in region III tended to decrease more than in region II. Here, the pressure under a rotation speed of 35 rpm was higher than that under a low rotation speed. This suggests that the desorption of crystallized water from gypsum is promoted under conditions of high rotational speed. The XRPD results in Figure 11 also show that the peak intensity of CaSO_4_∙0.5H_2_O increased as the rotation speed increased.

**Figure 12:**
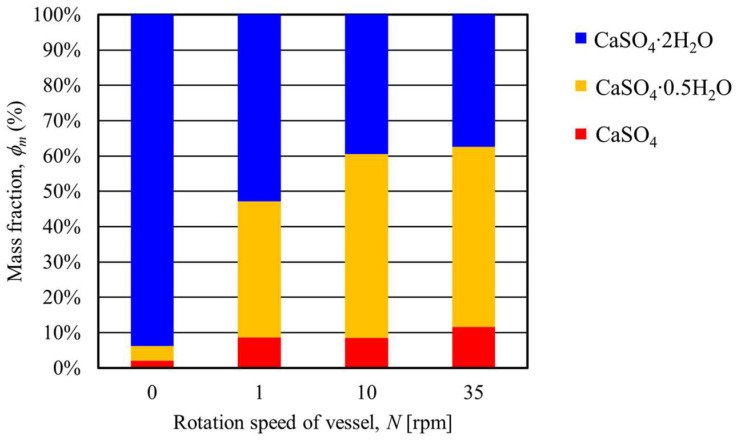
Relationship between the transferring mass fraction of gypsum and the rotation speed of the rotary vessel at a heating end temperature of 150 °C.

Table 3 shows the mass fractions of CaSO_4_∙2H_2_O, CaSO_4_∙0.5H_2_O, and CaSO_4_ when the heating end temperature was 150 °C and the rotation speed was varied. Figure 12 shows the results of the mass fraction of gypsum at each rotation speed. The figure shows that when the heating end temperature was 150 °C and the rotation speed was 1 rpm, the mass fraction of CaSO_4_∙0.5H_2_O was 38.58%, indicating that CaSO_4_∙2H_2_O was converted to CaSO_4_∙0.5H_2_O. Next, there was no significant difference in the mass fractions of CaSO_4_∙0.5H_2_O when the rotation speeds were 10 rpm and 35 rpm. On the other hand, these mass fractions of CaSO_4_∙0.5H_2_O increased by about 13% compared to the result of the rotation speed of 1 rpm. The above results show that the conversion from CaSO_4_∙2H_2_O to CaSO_4_∙0.5H_2_O is promoted by increasing the rotation speed.

**Figure 13:**
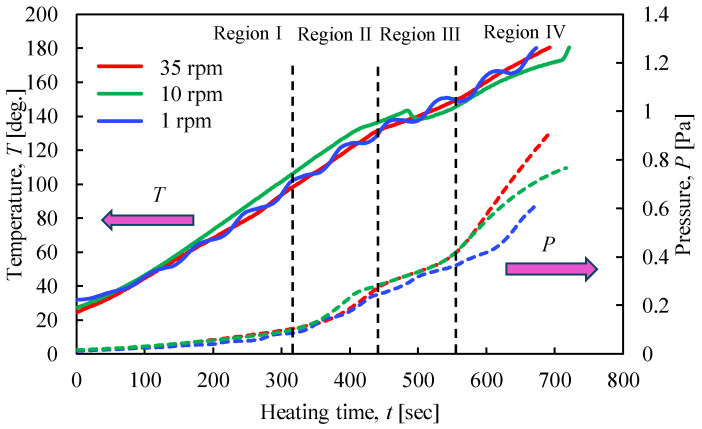
Time histories of temperature and pressure inside the rotary vessel at a heating end temperature of 180 °C when the rotation speed was changed.

**Figure 14:**
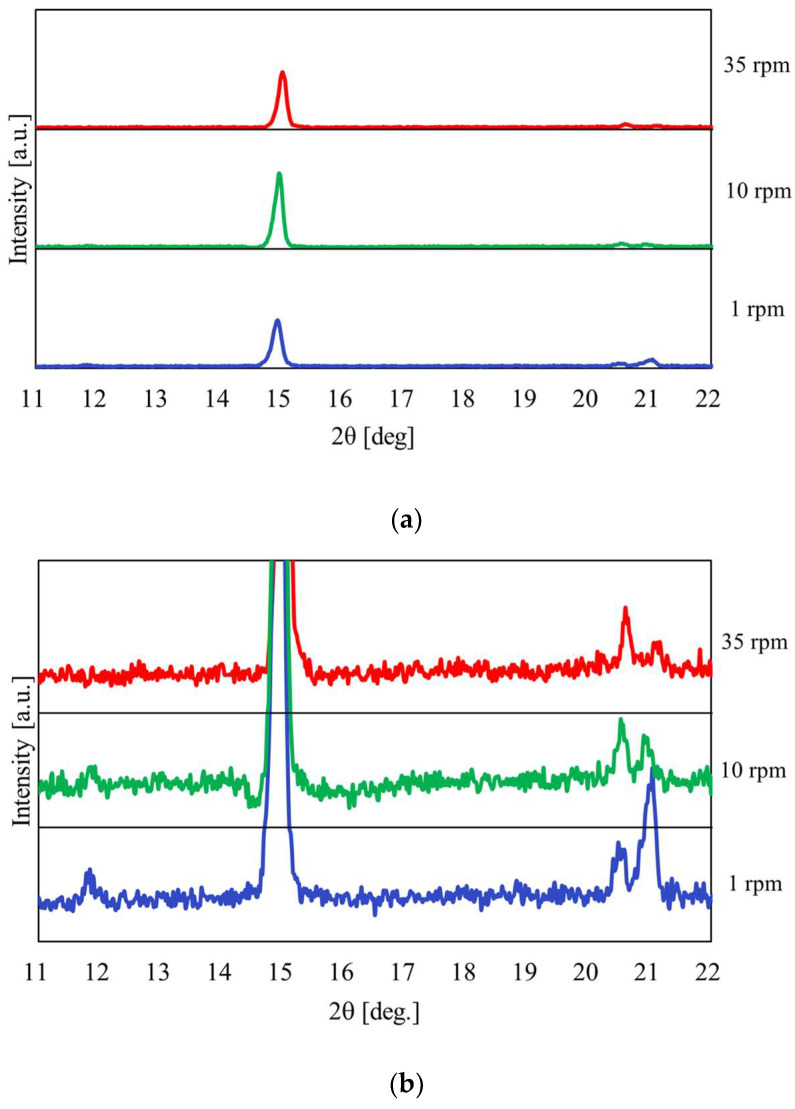
XRPD measurement results at a heating end temperature of 180 °C when the rotation speed was changed; (**a**) Normal figure; (**b**) Enlarged figure.

Figure 13 shows the relationship between the temperature and pressure inside the vessel concerning the heating time with the heating end temperature set at 180 °C. Figure 14 shows the results of the XRPD analysis at rotation speeds of 1, 10, and 35 rpm. In Figure 13, when the heating end temperature reaches 180 °C, the temperature and pressure gradients increase in region IV. In particular, the results show that the pressure increases rapidly as the rotation speed increases. As can be seen from the enlarged view of Figure 14b, under high rotational speed conditions, the peak intensity of CaSO_4_∙2H_2_O no longer exists, and the conversion from CaSO_4_∙2H_2_O to CaSO_4_∙0.5H_2_O is promoted.

**Figure 15:**
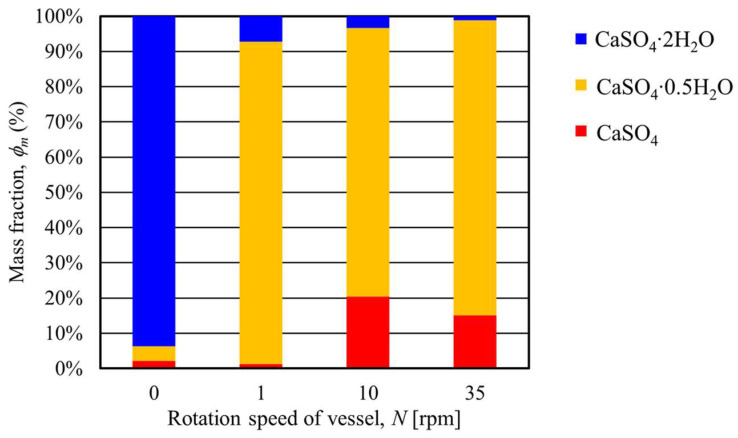
Relationship between the transferring mass fraction of gypsum and the rotation speed of the rotary vessel at a heating temperature of 180 °C.

Table 4 shows the mass fractions of CaSO_4_∙2H_2_O, CaSO_4_∙0.5H_2_O, and CaSO_4_ under conditions where the heating end temperature was 180 °C and the rotation speed was varied. Figure 15 summarizes the mass fractions of CaSO_4_∙2H_2_O, CaSO_4_∙0.5H_2_O, and CaSO_4_ at each rotation speed. As shown in Figure 15, when the heating end temperature was 180 °C and the rotation speed was 1 rpm, the mass fraction of CaSO_4_∙2H_2_O was 7.27% and CaSO_4_∙0.5H_2_O was 91.48%. This result shows that almost all CaSO_4_∙2H_2_O was converted to CaSO_4_∙0.5H_2_O under a rotation speed of 1 rpm. Furthermore, the 10 and 35 rpm results show a lower mass fraction of CaSO_4_∙2H_2_O than the result of the rotation speed of 1rpm. Here, under these conditions, the mass fraction of CaSO_4_ increased while CaSO_4_∙0.5H_2_O decreased. These results show that increasing the rotation speed promotes the thermal dehydration of gypsum and that CaSO_4_∙0.5H_2_O begins to be converted to CaSO_4_. The above results confirm that increasing the rotation speed accelerates the heat conduction and heat transfer of the particles in the vessel [13].

Here, the results of temperature distribution in relation to heating time will be considered. A wave-like periodic temperature change was observed at a rotation speed of 1 rpm. On the other hand, no frequent temperature changes were observed when the rotation speed was 10 rpm or higher. This difference in temperature change at different rotation speeds is related to the gypsum’s rotational flow inside the vessel. Therefore, we observed the flow pattern of the gypsum inside the rotating vessel.

**Figure 16:**
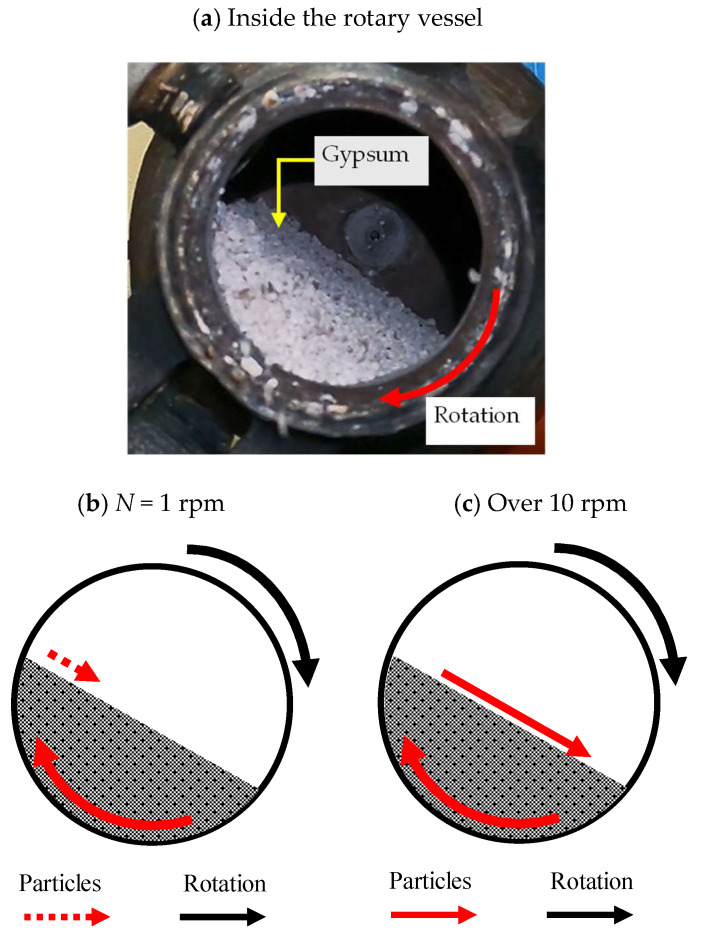
Typical flow pattern of gypsum particles in a rotating vessel.

Figure 16a shows a snapshot of the inside of the rotating vessel. Figure 16b,c show the schematic diagrams of particle flow based on Figure 16a. As a result, it was confirmed that in a rotating vessel, the gypsum rose along the vessel wall as the vessel rotated. After that, the particles collapsed and returned to the bottom of the vessel when they reached a certain height. Here, it was found that gypsum particle collapse occurred periodically under low rotational speed conditions, whereas under conditions over 10 rpm, it occurred continuously. It is thought that changes in temperature behavior appeared depending on differences in how the gypsum particles inside the vessel collapsed.

**Figure 17:**
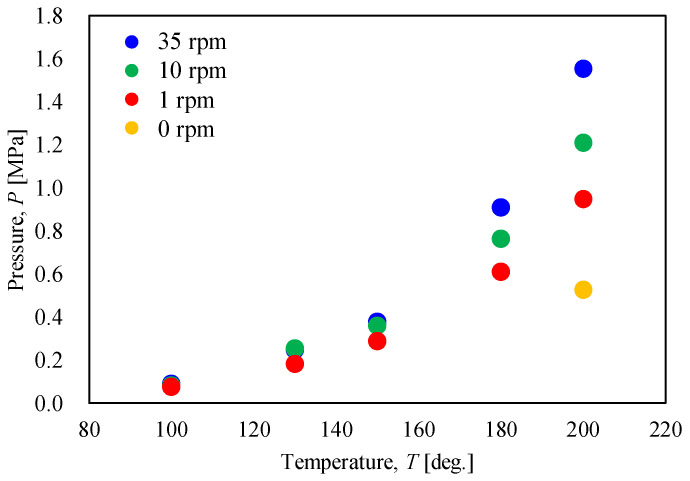
Relationship between the pressure in the rotary vessel and the heating end temperature when the rotation speed was changed.

**Figure 18:**
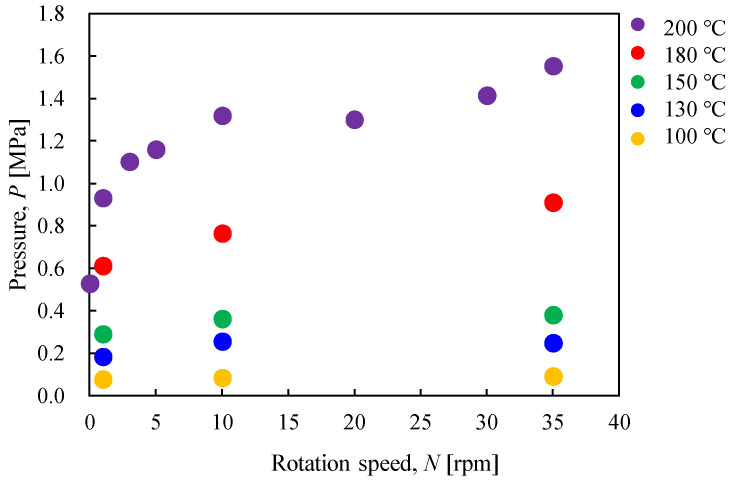
Relationship between the pressure in the rotary vessel and the rotation speed when the heating end temperature was changed.

Figure 17 shows the relationship between the internal pressure of the vessel and the heating end temperature when the rotation speed is varied. Furthermore, Figure 18 shows the relationship between the internal pressure of the vessel and the rotation speed at each heating end temperature. Both figures show that the ultimate pressure inside the vessel increased as the heating end temperature and rotation speed increased. This is because the rotation given to the vessel filled with gypsum promoted the flowability and heat conduction of the gypsum particles, leading to the active desorption of crystallized water. Also, as shown in Figure 18, the internal pressure of the vessel increased as the rotation speed increased until the rotation speed was around 10 rpm. On the other hand, when the rotational speed exceeded 10 rpm, the growth rate of pressure tended to decrease. This trend suggests a limit to the rotation speed for promoting thermal dehydration.

## 4. Conclusions

This study investigated the thermal dehydration characteristics of CaSO_4_∙2H_2_O using constant-volume rotary heating equipment. The results obtained are shown below.

(1) It was confirmed that the temperature distribution against the heating time changed due to the difference in the flow pattern of particles in a rotary vessel when the rotation speed was varied.
(2) Temperature and pressure distribution in regions I to IV depends on the detachment of crystallized water from the gypsum.
(3) It was found that the desorption of gypsum crystal water was promoted when the rotary vessel’s rotation speed was increased. As a noteworthy point, the pressure inside the vessel in region IV increased rapidly. The result indicates that the crystallized water was released from the gypsum.
(4) We obtained the conversion characteristics of CaSO_4_∙2H_2_O to CaSO_4_∙0.5H_2_O using a constant-volume rotary vessel. In the present heating equipment, the mass fraction of CaSO_4_∙0.5H_2_O increased when the temperature was 180 °C and rotation was applied.

## Figures and Tables

**Table 1:** Results of the mass fraction of CaSO_4_∙2H_2_O, CaSO_4_∙0.5H_2_O, and CaSO_4_ using Rietveld analysis at a heating end temperature of 100 °C when the rotation speed was changed.

*N* (rpm)	*T* (°C)	CaSO_4_∙2H_2_O *ϕ_m_* (%)	CaSO_4_∙0.5H_2_O *ϕ_m_* (%)	CaSO_4_ *ϕ_m_* (%)
0	0	93.74	4.14	2.13
1	100	87.30	6.18	6.52
10	100	89.49	6.96	3.56
35	100	85.44	8.44	6.22

**Table 2:** Results of the mass fraction of CaSO_4_∙2H_2_O, CaSO_4_∙0.5H_2_O, and CaSO_4_ using Rietveld analysis at a heating end temperature of 130 °C when the rotation speed was changed.

*N* (rpm)	*T* (°C)	CaSO_4_∙2H_2_O *ϕ_m_* (%)	CaSO_4_∙0.5H_2_O *ϕ_m_* (%)	CaSO_4_ *ϕ_m_* (%)
0	0	93.74	4.14	2.13
1	130	81.41	9.77	8.89
10	130	82.98	13.06	3.95
35	130	71.89	19.04	9.07

**Table 3:** Results of the mass fraction of CaSO_4_∙2H_2_O, CaSO_4_∙0.5H_2_O, and CaSO_4_ using Rietveld analysis at a heating end temperature of 150 °C when the rotation speed was changed.

*N* (rpm)	*T* (°C)	CaSO_4_∙2H_2_O *ϕ_m_* (%)	CaSO_4_∙0.5H_2_O *ϕ_m_* (%)	CaSO_4_ *ϕ_m_* (%)
0	0	93.74	4.14	2.13
1	150	52.87	38.58	8.66
10	150	39.41	51.91	8.56
35	150	37.39	51.01	11.60

**Table 4:** Results of the mass fraction of CaSO_4_∙2H_2_O, CaSO_4_∙0.5H_2_O, and CaSO_4_ using Rietveld analysis at a heating end temperature of 180 °C when the rotation speed was changed.

*N* (rpm)	*T* (°C)	CaSO_4_∙2H_2_O *ϕ_m_* (%)	CaSO_4_∙0.5H_2_O *ϕ_m_* (%)	CaSO_4_ *ϕ_m_* (%)
0	0	93.74	4.14	2.13
1	180	7.27	91.48	1.25
10	180	3.35	76.19	20.45
35	180	1.14	84.00	15.20

## Data Availability

Data are contained within the article.
